# Mental health and post-traumatic growth in multiple sclerosis

**DOI:** 10.1192/j.eurpsy.2023.675

**Published:** 2023-07-19

**Authors:** I. Gil-González, M. Á. Pérez-San-Gregorio, A. Martín-Rodríguez

**Affiliations:** Department of Personality, Assessment, and Psychological Treatment, University of Seville, Seville, Spain

## Abstract

**Introduction:**

people suffering from multiple sclerosis (MS) can experience post-traumatic growth (PTG), a sense of personal growth and benefit gain. Patients mental health can play an important role in PTG development.

**Objectives:**

to explore possible differences in mental health according to PTG levels.

**Methods:**

the sample was composed of 392 outpatients with MS from Virgen de la Macarena University Hospital (268 women; 124 (31.6 %) men, ages 19-78 years old (mean 45.61 years, SD=11.16 years). Expanded Disability Status Scale (EDSS) mean score was 3.38 (SD=2.06). Relapsing remittent (n=327) and progressive (n=65) MS type were reported. Post-traumatic Growth Inventory (PGI-21) measured patients perception of personal benefit gain after MS experience. General Health Questionnaire-28 (GHQ-28) evaluated Mental Health distress symptoms. Unpaired t-test was used to identify differences in mental health distress between “low PTG ≤49 score” and “high PTG ≥50 score” groups.

**Results:**

Significant differences were found in social dysfunction (t=2.521, p=0.012) and severe depression (t=2.442, p=0.015), “high PTG group” (n=194) presented lower scores compare to “low PTG group” (n=198). No significant difference was detected in somatic symptoms (t=0.185, p=0.087) and anxiety and insomnia (t=0.859, p=0.391).

**Conclusions:**

patients with higher PTG reported a better mental health. This suggests the relevance of mental health status in positive outcomes development after an adverse life event. Particularly, social dysfunction and depressive symptoms should be considered in interventions aimed to promote positive outcomes as personal gain and benefit finding in MS population.

**Disclosure of Interest:**

None Declared

